# Linking people with long‐term health conditions to healthy community activities: development of Patient‐Led Assessment for Network Support (PLANS)

**DOI:** 10.1111/hex.12088

**Published:** 2013-06-03

**Authors:** Christian Blickem, Anne Kennedy, Ivaylo Vassilev, Rebecca Morris, Helen Brooks, Praksha Jariwala, Tom Blakeman, Anne Rogers

**Affiliations:** ^1^ Centre for Primary Care, Institute of Population HealthUniversity of ManchesterManchesterUK

**Keywords:** long‐term conditions, patient and public involvement, self management, social networks, social prescribing, voluntary and community groups

## Abstract

**Objective:**

To combine insights from service users with long‐term conditions (LTCs) to assist the development of a community referral intervention designed to promote engagement and improve access to health‐relevant resources.

**Background:**

Social deprivation and reduced access to resources have been causally linked with social isolation and the ability to manage LTCs. Participation in meaningful activity has been associated with positive health benefits, and strategies to promote access to community activities have shown some potential to improve outcomes for people with LTCs. This suggests the need to develop an engagement and referral intervention in partnership with service users and community groups as part of mainstream self‐care support.

**Method:**

A series of focus groups and interviews with members of community groups in Greater Manchester designed as an iterative and collaborative approach to elicit the role of personal and community networks that support long‐term condition management (LTCM) to develop a community referral tool.

**Results:**

Participants reported a broad range of resources relevant to LTCM that often went beyond the usual concerns associated with self‐care. This helped to inform a tool (PLANS) to tailor access to types of community‐based resources which can support LTCM.

**Conclusions:**

Understanding the everyday challenges of living with a LTC highlighted the importance of connecting and engaging with localized support for people. In response to this, we developed an intervention (PLANS) which tailors access to local resources based on personal preferences, needs and acceptability to encourage service users to engage with sustainable health choices.

## Introduction

Long‐term conditions (LTCs) are the leading cause of ill health and disability in the UK^1^, ^2^ and are disproportionately experienced by socially deprived groups who suffer most from reduced access to resources and social isolation. Building on evidence that demonstrates clear links between social isolation and chronic illness,^3^, ^4^ this article outlines the development of a strategy to identify need and promote engagement of a tool to facilitate links to health‐relevant resources for people with LTCs.

A focus for people with LTCs is self‐care support to increase capacity, confidence, efficacy and improve knowledge and personal skills of individuals.^5^, ^6^ However, there is equivocal evidence regarding the outcomes of existing programmes for socially disadvantaged groups,^7^, ^8^, ^9^, ^10^, ^11^, ^12^, ^13^ begging the question of the extent to which context and access to a broad range of resources necessary for living life with a LTC can be included within the remit of self‐care support. A pre‐occupation with a focus on individual behaviour change implies the merits of a shift in emphasis to consider the role and use of community and networks, which to date has been unacknowledged.^14^ Developing strategies of support for people with LTCs within everyday settings which allow for social and structural factors to be taken into account potentially complements provision in primary and secondary care.

## Resources for self‐care support: beyond the individual

Social prescribing offers one model for utilizing voluntary and community support by promoting access to locally available health groups (e.g. weight management, exercise).^15^ This indicates the possibilities of creative engagement between primary care and non‐traditional providers of health care (NTPs) as the basis for attempts to develop dedicated tools.^15^, ^16^, ^17^, ^18^ Evidence of patient outcomes from one study reported a reduction in isolation, increased confidence, improved access to non‐stigmatized support, improvement in the patient–clinician consultation and reduced clinician workload.^19^

Despite the potential that social prescribing offers, it has had limited impact due to lack of evidence, scepticism by clinicians, the complexities of establishing and sustaining a database of local resources and the absence of a logical process of referral of people to appropriate local resources.^15^, ^16^, ^18^ The latter suggests the need for intervention development and empirical work to explore the barriers and enablers to accessing support and to identify the types of support people with LTCs most value. The ethos of ensuring that support becomes a normal part of people's day‐to‐day activities is relevant for maximizing the likely uptake and embeddedness of an intervention designed to link people up with resources and networks.^20^, ^21^ In this article, we describe the process of identifying the meaning and role of the community and voluntary sector for people with LTCs, the development of an intervention, and ways of working with people to develop a strategy for linking people to local support.

## Patient and public involvement and normalization process theory

That the development of complex interventions should involve engagement with service users has become increasingly normative^22^, ^23^ with a number of studies demonstrating the value of incorporating the views of service users.^24^, ^25^, ^26^ There is increasing evidence that involving lay participants in intervention development has the potential to bridge understanding between the clinical and everyday experiences of people with LTCs.^17^ However, the practical means of engagement are currently underdeveloped and inconsistent, and the development processes have been critiqued for being opaque, rarely involving negotiation with lay members of the public and removed from the everyday contexts of peoples' lives.^17^

Moreover, patient as well as clinical interventions are liable to fail because they pay insufficient attention to the necessary conditions of implementation at the development stage of new interventions. Normalization Process Theory is an implementation theory and helps in providing awareness of the work involved in embedding and sustaining practices associated with an intervention, and thus optimizing the chance of becoming normalized into everyday settings.^20^ Drawing on four constructs,^1^ normalization process theory (NPT) attempts to understand the uptake and embedding of an intervention with reference to judging how a new tool is likely to impact on interactions between people and practices; how this relates to people's existing knowledge relationships; the division of labour; and the organizational and other settings in which it is set. In the arena of self‐management support, the interface between lay and clinical is of most salience.^14^ We have used NPT to guide the development of PLANS in a way in which incremental changes could be made on the bases of feedback at different stages from patients, and with reference to the technological, primary care and community settings, the tool was orientated to operate within. Of particular, salience is patient normalization. That is, to be an optimal candidate for normalization, a new tool (such as the one proposed here) should seek a ‘fit’ with the actual or realizable set of roles within patients' division of labour and be capable of *integration* within existing or realizable patterns of self‐management and service contact with professionals. It follows from this that the advantage to patients must be tangible and evident to their everyday illness work and contact with services is crucial to the evaluation of new interventions and practices.

Thus, informed by NPT we outline the development of a community referral tool (PLANS) for people with LTCs in partnership with lay members. We describe the development of the tool using focus groups and interviews and illustrate how we tried to reflect the concerns and everyday life support needs raised.

## Methods

Ethics approval for the study was granted by the North West Greater Manchester Central Ethics Committee (ref: 10/H1008/1). All participants provided written informed consent at the start of their involvement.

The methods used to develop and pilot the PLANS tool as an interactive website involved a two‐stage process: obtaining initial grassroots understanding about the use of localized support and then involving service users in the subsequent piloting and evaluation.

**Table:** 

Stage 1	Five focus groups with established community groups in Greater Manchester	Exploring the meaning and role of the community and voluntary sector for people with LTCs. Development of prototype PLANS
Stage 2	Six participatory workshops with a PPI group of service users with links to the groups in stage 1 Eight interviews with members of our PPI group	To refine and pilot an early prototype PLANS tool and gather feedback about practical implementation

### Stage 1: exploring the meaning and role of the community and voluntary sector for people with LTCs

This stage was intended to gather a broad range of perspectives on the meaning and role of the community and voluntary sector for people with LTCs. We recruited a convenience sample of people from health‐related support groups and community centres offering a variety of activities, for example exercise, hobbies and interests. These groups were selected on the basis that they had local memberships and provided activities or services which were relevant to health or well‐being and were in areas of high deprivation. We purposefully selected members from these groups to represent a range of conditions, ethnicity, gender and age.

Three researchers conducted each focus group, and the sessions were held in the groups' usual setting (See Table 1 for participant demographic characteristics). To prompt discussions, we used an amalgam of personal narratives of people living with a long‐term condition with opportunities for participants to comment on other's experiences and then interject with their personal thoughts and experiences.^27^, ^28^ These groups were audio‐recorded and lasted between 1 and 2 h.

**Table 1:** Demographics of participants in the stage 1 and 2 focus groups

	ID	Type	Number	Gender	Ethnicity
Stage 1 focus groups	1	Cardiac support group	5	All male	All white
	2	Diabetes support group	8	Mixed	All white
	3	Sugar group	6	All female (a women's group)	All Afro Caribbean
	4	Good Neighbours group	10	Mainly female (one male)	Mixed
	5	Community centre group	5	All male	All white
Stage 2 focus groups	6	Mixed from stage 1	6	Mixed	Mixed

### Stage 2: developing, refining and piloting the PLANS tool

To test the acceptability and usability of the tool, we conducted six participatory workshops with members of our Patient and public involvement (PPI) group recruited from stage 1 (these were not audio‐recorded, but comprehensive field notes were taken by a researcher). We then conducted eight interviews with people with LTCs recruited from existing contacts, who had agreed to participate in our PPI work to refine the intervention and provide feedback about practical implementation (details of the PLANS tool are described in the analysis section, stage 2 and a final version can be found in Table 3). This stage included a related resource called CONECTS (Community and Networks for Condition Support), which is a series of short films about the experiences of two people with vascular disease and the difficulties they have managing their health and who have tried taking part in community activities (specifically walking and slimming groups). These films were shown to participants in the focus groups to encourage reflections on their experiences of engaging in social and community activities. These sessions lasted around 2 h.

For the qualitative interviews, we used a ‘think aloud’ method that focuses on respondents verbalizing their thoughts and decision making during a task.^29^ This method was used as a way to conduct a detailed exploration of the way participants understood and responded to PLANS and to better understand how the intervention might improve awareness of local support and participation in activities that have health benefits. The ‘think aloud’ interviews were followed by semi‐structured interviews allowing reflection about the process and the influence of PLANS on how people feel about accessing local groups and support (See Table 2 for participant demographic).^30^, ^31^ The interviews generally lasted between 30 and 45 min and were audio‐recorded.

**Table 2:** Demographics of participants of stage 2 interviews

Person ID	Sex	Age	Ethnicity	Location
P1	M	70's	White	Oldham
P2	F	70's	White	Oldham
P3	M	50's	White	Oldham
P4	F	70's	White	Oldham
P5	M	50's	White	Bolton
P6	M	50's	White	Bolton
P7	M	70's	White	Levenshulme
P8	M	50's	White	Levenshulme

### Analysis

All authors contributed to three rounds of analysis and discussions where a consensus was reached on key topics. Each author read at least two transcripts with associated field notes and listened to two audio recordings of the focus groups and interviews. All authors contributed to analysis discussions where a coding scheme was developed and refined. The focus groups and interview data were coded thematically^32^ by CB and PJ and discussed with the research team at analysis meetings. Themes were identified whilst allowing the stories and the context in which they occur to be examined and category consensus reached, leading to the emergence of several themes related to the concept of PLANS.^33^ For the analysis of the focus groups in stage 1, themes were developed into categories for the PLANS tool and reviewed by the research team. Analysis of the focus group and interviews in stage 2 provided further insights into the themes and categories developed in stage 1. Thematic analysis was conducted by CB and PJ, and discussions with the research team informed a further important theme (mobility). This process was conducted until category saturation was reached. CB and PJ used the coding framework to analyse the qualitative interviews to evaluate and refine the PLANS tool.

#### Stage 1: findings from community focus groups

Topics raised in the focus groups gave some insight into the complementary and alternative functions of community groups with regard to self‐care, the meaning and the role of the community and voluntary sector for people with LTCs, and the types of support people with LTCs found valuable and how they can be found within the local community. Thematic analysis identified three principal themes: isolation, safety and linking to support; the group's power to normalize the problems of chronic illness; reciprocal communities, namely groups as a forum for exchange of emotional and practical support. The following section explores these themes with the aim of highlighting the role of community and voluntary groups in supporting people with LTCs to manage their health.

##### Isolation, safety and linking to support

All participants felt that the groups they attended played an important social function in their lives, as many had reduced social contact due to retirement, limited mobility, finances or because they had lost their partner/spouse. It emerged that loneliness and isolation was for many the most difficult part of getting older or coping with poor health, and attending their group was a rare opportunity for social contact. For example,

I came here because I retired in 2008, it was wonderful for the first few weeks, I didn't have to get up in the morning, I could lie in. But as time goes on you start to get bored ‐ depression sets in ‐ so I said I've got to get out of this rut. So I went to my doctor and he says ‘go and join (this group)’ and things like that, which I've found very very helpful.

(Male, 70s, 4)

The groups were described as a ‘safe place’ where members could share meals with others or engage in social interaction, but also served other functions that were initially less recognized by participants. For example, some participants who lived alone told how their group provided security, for example if they were absent from the usual events, then someone would contact them to ask how they were. Groups also provided an access point to a range of everyday support such as transport, home help or advice about benefit entitlement. Awareness of these resources was generally limited to links through the groups. The only other alternative was the GP who was not regarded as appropriate to perform this function. Linkage to these resources through the groups was described as a lifeline to help which otherwise participants struggled to know how to access. Because these types of support were for seemingly trivial things such as odd jobs around the house, participants were unlikely to actively seek help. However, in the context of the group, these concerns were more easily shared with others who had similar experiences.

##### The group's power to normalize the problems of chronic illness

The groups provided opportunities to participate in a variety of activities that had more direct links to health such as exercise groups, but significantly these were talked about as part of a variety of social activities on offer. For example:

We came to do paintings first and er, it developed into all sorts of things, all different kind of paintings and er, and then we, we, we come up to the exercise class, and er, one of the main reasons is you're meeting with other people; you're not getting bored, and er, well it's so kind in this place and they help you as much as they can.

(Female 70s, 4)

The exercise classes appeared to be tailored to suit the range of mobility restrictions of the group and importantly they seemed situated within a familiar and comfortable environment where participants were doing enjoyable, everyday things. Hence, familiarity with the surroundings and the other people involved appeared to create an encouraging atmosphere for participants to take part in exercise. Similarly, all the groups gave participants a chance to discuss topics related to lifestyle and health with other people in similar circumstances in a relaxed and supportive atmosphere where they could share tips or vent frustrations. The rhetoric of these forums was occasionally defiant of clinical guidance which some felt was at odds with meeting everyday life challenges. This seemed to be an important process towards achieving a personally acceptable long‐term condition management (LTCM) plan and links with the overall notion of community groups as a form of self‐care support which is not available through formal or usual channels.

##### Reciprocal communities

Furthermore, giving a sense of purpose to the day and having something to look forward to attending the groups offered members an opportunity to play a valued social role. For example, one participant said,

I do all the minor repairs in the church…I enjoy it, working for people, helping people…

(Male 80s, 5)

Members undertook tasks for the group such as delivering newspapers, preparing food or helping with form filling. Participants in the focus groups were very keen to stress their active involvement in what they described as a ‘community’. Feeling valued and doing things for others appeared to be at least as important to members as receiving support. This contrasts sharply with the type of formal self‐care support available that generally requires an individual focus and passive acceptance of clinical and lifestyle advice. The groups seemed to provide an informal setting in which member had the chance to access a range of social or practical resources which was reciprocated by the members. This active engagement with the group was a significant motivating factor for many of the members to be positive about themselves, their lives and their health.

#### Stage 2: developing and piloting PLANS: Findings from participatory workshops

Drawing on these findings and the literature, the focus group analysis informed the development of a tool designed to improve awareness of existing local resources and make clear links to local support based on the criteria of expressed ‘need’ and ‘acceptability’, which we call PLANS. The idea of PLANS is to reflect the everyday needs and concerns of people who live with a LTC and consolidate up‐to‐date information about health‐relevant local resources into one website. The website contains a self‐assessment questionnaire, which is completed by users who are provided with a tailored set of options based on personal preference. The types of support people with LTCs benefit from as derived from the focus groups included the following:

1. Opportunities for meaningful and enjoyable things: well‐being.
2. Access to personally relevant information about health problems: health education.
3. Help with everyday practical problems and access to a range of local services to support independent living: practical support.
4. Access to locally available and affordable activities to help with exercise and healthy eating: diet and exercise.

We then conducted an internet search for these types of support in an area of Greater Manchester (where we intended to conduct further workshop groups) to (i) ascertain active local groups (ii) inform the development of a typology of groups, services and support and (iii) create a website and database with tailored links to local groups, services and support. We used the following search terms together with ‘Oldham, Greater Manchester’ (Box 1):

Box 1 **PLANS categories and search terms**

**Table:** 

Categories	Search terms
Well‐being	Social activities; hobbies; counselling
Health education	Diabetes; kidney disease; heart disease
Practical support	Home support; independent living
Diet	Weight management; healthy eating
Exercise	Gym; keep fit; leisure centres

We developed a short questionnaire measuring (i) *Need,* for example what health‐related problems are reported by users and (ii) *Acceptability,* for example what solutions are locally available that match with things people like and can do. We adapted the questions from closely matched items in the Health Education Impact Questionnaire (heiQ) to encapsulate the key concepts and themes from our focus group findings.^34^ We chose the heiQ as an exemplar because its development took a similar grassroots approach. Development of the heiQ involved extensive work with patients and other stakeholders to target crucial outcomes of patient education programs for people with chronic disease. We then added Likert‐style scales so that if the user chose either ‘disagree’ or strongly disagree’, then they were directed towards the corresponding category option (Table 3).

**Table 3:** Final version of the PLANS tool

Category of support	Item definition	Description of group/activity	PLANS question *NEED* a					PLANS subcategories *ACCEPTABILITY* b choose from the following options
CONECTS (Community and Networks for Condition Support)	Normalisation (May)	Online resource of short films of patient's experiences of managing with long‐term health problems and balancing everyday life.	I find that my illness is making life difficult for me					Online resource
			Strongly Agree	Agree	Neither Agree or Disagree	Disagree	Strongly Disagree	
			(Scale is reversed so if ‘strongly agree’ or agree’ user is directed to the CONECTS category)					
Health education	HeiQ Self monitoring and insight (Osborne 2007)	Groups, services and activities that offer health‐related advice, guidance and support	I feel I know a lot about my condition					Healthy eating groups Education about your health
			Strongly Agree	Agree	Neither Agree or Disagree	Disagree	Strongly Disagree	
Practical support	HeiQ Social integration and support (Osborne 2007)	Groups, services and activities that offer everyday practical support	I am in contact with many people who can help and support me					Advocacy and advice groups Befriending services Day care and adult respite services Education and training services Home support services
			Strongly Agree	Agree	Neither Agree or Disagree	Disagree	Strongly Disagree	
Diet	HeiQ Health‐directed behaviour (Osborne 2007)	Groups, services and activities that offer support and guidance for diet and healthy eating	I am coping very well with shopping, cooking and things related to my diet					Weight management groups Healthy cooking/eating groups
			Strongly Agree	Agree	Neither Agree or Disagree	Disagree	Strongly Disagree	
Exercise	HeiQ Health‐directed behaviour (Osborne 2007)	Groups, services and activities that provide opportunities to keep fit and participate in physical activity	I am happy with the opportunities I have to be active, for example walking and doing exercise					Sport and gyms Fitness and exercise classes Swimming pool activities Martial arts and combat groups Walking and outdoor activities Rehabilitation groups
			Strongly Agree	Agree	Neither Agree or Disagree	Disagree	Strongly Disagree	
Wellbeing	HeiQ Positive and active engagement in life (Osborne 2007)	Groups, services and activities intended for general wellbeing and social participation.	I am happy with opportunities to participate in social activities or other things I enjoy					Literature and writing groups Arts and craft groups Drama and music groups Hobbies and interest groups Counselling services Other social activities
			Strongly Agree	Agree	Neither Agree or Disagree	Disagree	Strongly Disagree	
Mobility	PPI development work	Services that offer support for people with limited mobility or have difficulty using public transport, for example shopping and delivery services	I find it easy to get to places I want to go					Shopping and delivery services Community transport services
			Strongly Agree	Agree	Neither Agree or Disagree	Disagree	Strongly Disagree	

a The health‐related problems reported by users.

b Locally available solutions that match with things people like doing.

The prototype PLANS website consisted of two stages of questions, the first stage measuring ‘need’ and the second ‘acceptability’. Once completed, users arrive at a set of results of groups/services including contact details, descriptions of activities and services which might be relevant and acceptable to them based on their answers to the questionnaire.

### Piloting the PLANS tool

Participants in the workshops were keen critics of the types of self‐care support available for people with LTCs and at times discussions tended to fixate on problems with medication and frustrations about encounters with medical professionals. There was general support for the PLANS tool, and by the end of the sessions, participants had each completed a questionnaire, and some members had even made independent enquiries about the groups in their PLANS results. These sessions appeared to encourage participants to consider the PLANS options because they offered space to reflect on barriers and facilitators to trying new things. The workshops provided further insights into the types of support people with LTCs value and some of the everyday barriers to accessing appropriate support. Analysis of notes from these workshops produced three key themes in relation to utilizing community resources for LTCM: previous experiences of groups; mobility; and existing relationships.

### Previous experiences of groups

Discussions became quite animated when participants talked about groups they had previously participated in. Some felt nervous about the idea of joining new groups, and others were reluctant to entertain the idea because of previous negative experiences of groups and dominant personalities or cliques. This is where the notion of prescribing activities for individuals became awkward as participants were initially resistant to the idea of being directed to attend a group. However, having an opportunity to vent some of their irritations about past experiences or anxieties seemed to clear the air and support engagement with their PLANS results.

#### Mobility

One of the major barriers to accessing local resources was mobility or lack thereof. Most participants did not have their own transport and so relied on public transport or family members if available. All of the participants lamented the expense of public transport and bus services unsuitable for people with restricted mobility. For many, this was a fundamental barrier to being actively involved in things they enjoyed. It was also noted that deprived communities will likely have fewer community resources and so access beyond the immediate area is important for those wanting to engage in community activities. Therefore, from a PLANS perspective, accessibility, transport and resources should be addressed by creating direct links to practical support.

#### Existing relationships

It became clear that awareness and information concerning community groups and resources are sporadic and that accessing resources relies on existing relationships or links within the community. Participants often found out about other groups or resources from their friends and family or from other groups they attended, exacerbating the divide with the isolated.

Limitations and difficulties with transport and access influenced engagement with community activities, and so, the category ‘mobility’ was added to the PLANS website and database (Table 3). The PLANS approach aims to make use of and create connections to community‐based activities as a normal part of everyday life. Getting people to reflect on barriers such as finding activities and anxiety about meeting, new people was a valued exercise during the workshops. Therefore, a further category CONECTS (with links to the short films mentioned previously) was added to the questionnaire and website with the aim of encouraging reflection on the difficulties of finding activities and attending groups. Questions were then formulated to capture these categories of support (Table 3).

### Piloting the intervention using ‘think aloud’ and qualitative interview techniques

Here, we summarize key findings from the interviews with a summary of guidance to support the delivery of PLANS (Box 2):

Box 2 **Findings from ‘think aloud’ interviews**

**Table:** 

Findings	Guidance for delivery of PLANS
Participants found the PLANS questions understandable.	Users can benefit from reflecting on activities they have performed in the past to help guide them towards options they may find acceptable.
Too much information about each option can be off‐putting. A short bullet‐point summary of each of the resources is user‐friendly.	Encourage users to think about the potential benefits of using a resource and about who is around them to help with things they want to do.
All participants felt that their results were useful andrelevant to them. Some felt that a conversation with a support worker to discuss the PLANS options would be helpful, and a written summary of the results would encourage them to take up some of the activities.	Highlight any problems with cost, transport, time or location and try to guide users to relevant PLANS categories (e.g. MOBILITY or practical support) for possible solutions.
During the interviews, it seemed helpful for participants to reflect on things they did in the past as these are the activities they are probably more likely to try again.	Encourage users to make a plan of action if they have decided they would like to try an activity. Detail day, time, transport and who might go with them.

## Discussion

The conceptual focus of PLANS builds on the notion that the needs of people with LTCs cannot be adequately met through small targeted interventions which are unrelated to everyday life. The great majority of these self‐care resources would be professionally‐led, individually centred, and prioritize clinical knowledge, formal narratives, adherence to medical advice and action planning. Whilst the value of this more traditional approach may be the preferred choice of some people with LTCs, we argue that parallel complementary health‐related resources need to be recognized as they play an important role in LTCM and are relevant to a broader group of people; particularly those living in deprived areas who would benefit from knowledge about what is locally available. Such an approach gives scope to align LTCM with everyday life priorities and personal preferences. This allows for a better understanding of the work of individuals and their networks in building individual and collective repertoires of health‐relevant practices. These repertoires could be nurtured through engagement with a range of health‐relevant resources which might already be part of a personal community or be locally available. Therefore, the key objective and outcome of our approach in researching and producing the PLANS tool has been to identify health‐relevant localized support and key parties and resources that might be implicated in the access and utilization of appropriate support.

Many participants in our study reported difficulties in staying active and involved in things around them because of the isolating effects of poor health and old age which is consistent with the literature about the associations between social deprivation, isolation and long‐term health problems.^1^, ^3^, ^4^ Therefore, a tool to increase social contact and promote community support and engagement within deprived populations has potential to address some of these factors and hopefully reduce the impact of social deprivation. The close engagement with people with LTCs during the development of PLANS helped inform a grassroots understanding of the range of health‐relevant support which is valued and locally available. Working sensitively with the concerns and priorities of people living with LTCs has significant potential to improve the effectiveness of health‐care campaigns in general.

Our empirical work and pragmatic application is grounded in and builds on established evidence and theory. The short PLANS questionnaire is based on normalization process theory by creating links to localized and personally relevant support for people with LTCs so that it becomes a normal part of people's day‐to‐day activities^20^, ^21^ and draws on work by Osborne and colleagues.^34^ Our PPI approach ensured we included pragmatic factors of key importance to our target group such as mobility. Appreciation of the everyday non‐clinical challenges that people with LTCs face is a core feature of the PLANS approach which aims to normalize LTCM by weaving together health and everyday life priorities so that LTCM sits more easily amongst the things they value and want to do with their lives. This approach has potential to increase the likelihood that users may utilize these resources because of their location and their associations with everyday life. Further financial benefits are possible by reducing the duplication of public services by the NHS and other state agencies.

The sustainability for PLANS would be enhanced if websites and databases maintained by organizations such as local councils, local authorities and the voluntary sector are utilized. In fact, one of the added values of PLANS is in highlighting the health benefits of locally available resources and the improved accessibility and relevance PLANS offers to these websites for people with LTCs. What is more, PLANS could be used as a part of an assessment of the availability and geographical spread of health‐relevant resources in specific areas and could therefore also inform the commissioning process.

It must be acknowledged that the approach we have developed here may have limitations, and indeed, PLANS is likely to work better in areas where the existing provision of suitable resources and community groups is well developed. It also cannot be taken for granted that existing groups will always welcome added exposure or new members. Furthermore, PLANS could also be expected to perform better if a part of a complex intervention aimed at addressing different aspects of improving community engagement rather than when used on its own. These limitations, however, only emphasize the complexities involved in shifting the emphasis in health provision away from a focus on individuals towards social engagement, well‐being and network support.

## Note

1 Interactional workability, relational integration, skill‐set workability and contextual integration.
